# Supplementary material for: The causal role of circulating inflammatory markers in osteoporosis: a bidirectional Mendelian randomized study
Source: Front Immunol. 2024 Jul 18;15:1412298. doi: 10.3389/fimmu.2024.1412298 (PMC11291241; doi:10.3389/fimmu.2024.1412298)
Supplement: Supplementary file 1 [file DataSheet_1.zip › supplementary material/OSTEOPOROSIS-CIM/GCST90274766.tsv.gz_forest.pdf]

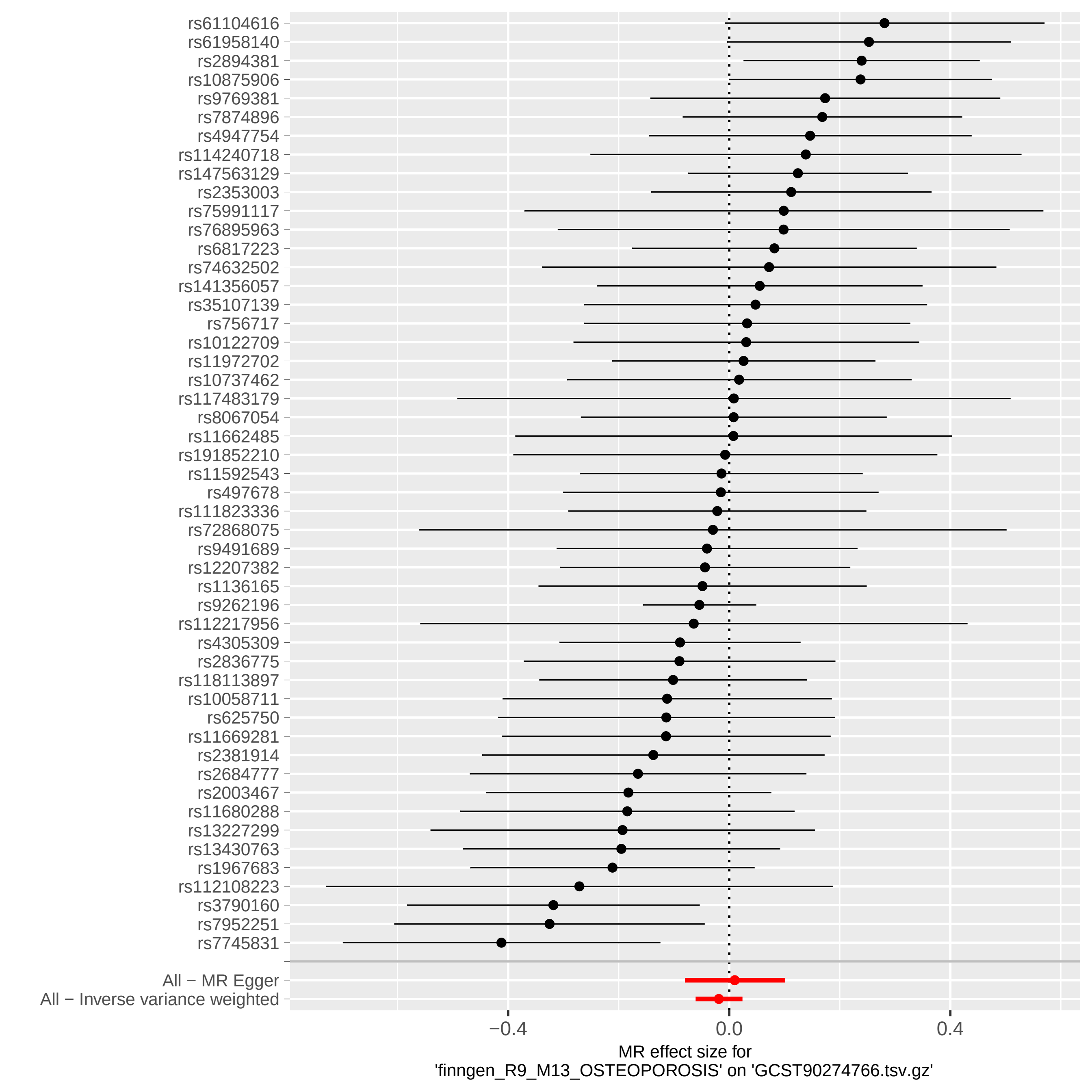

rs61104616
rs61958140
rs2894381
rs10875906
rs9769381
rs7874896
rs4947754
rs114240718
rs147563129
rs2353003
rs75991117
rs76895963
rs6817223
rs74632502
rs141356057
rs35107139
rs756717
rs10122709
rs11972702
rs10737462
rs117483179
rs8067054
rs11662485
rs191852210
rs11592543
rs497678
rs111823336
rs72868075
rs9491689
rs12207382
rs1136165
rs9262196
rs112217956
rs4305309
rs2836775
rs118113897
rs10058711
rs625750
rs11669281
rs2381914
rs2684777
rs2003467
rs11680288
rs13227299
rs13430763
rs1967683
rs112108223
rs3790160
rs7952251
rs7745831
All − MR Egger
All − Inverse variance weighted
−0.4
0.0
0.4
MR effect size for
'finngen_R9_M13_OSTEOPOROSIS' on 'GCST90274766.tsv.gz'
